# Ocular *Spiroplasma ixodetis* in Newborns, France

**DOI:** 10.3201/eid2602.191097

**Published:** 2020-02

**Authors:** Alexandre Matet, Anne Le Flèche-Matéos, François Doz, Pascal Dureau, Nathalie Cassoux

**Affiliations:** Institut Curie and Université Paris Descartes, Paris, France (A. Matet, F. Doz, N. Cassoux); Institut Pasteur, ​Paris (A. Le Flèche-Matéos);; Fondation Ophtalmologique Adolphe de Rothschild, Paris (P. Dureau)

**Keywords:** Spiroplasma, uveitis, cataract, eye, infection, microphthalmos, infant, newborn, PCR, bacteria, France, ticks, vector-borne infections

## Abstract

Cataract and uveitis are rare in newborns but potentially blinding. Three newborns with cataract and severe anterior uveitis underwent cataract surgery. *Spiroplasma ixodetis* was detected in lens aspirates using bacterial 16S-rRNA PCR and transmission electron microscopy. These findings, which suggest maternal–fetal infection, are consistent with previous experimental *Spiroplasma*-induced cataract and uveitis.

*Spiroplasma* is a genus of Mollicutes, a class of bacteria without cell wall. *Spiroplasma* are intracellular organisms with helical morphology and a small genome (0.78–2.2 Mb) comprising 38 species isolated from insects, crustaceans, and plants (*1*,*2*). Ticks, from which *Spiroplasma ixodetis* has been isolated, are abundant sources of *Spiroplasma* (*3*). 

*Spiroplasma* develop a commensal, pathogenic, or mutualist pathogen–host relationship. The first isolated species (*S. citri*) was described in 1973 (*4*,*5*). Two *Spiroplasma* infections have been reported in humans: an intraocular infection in a newborn with Group VI *Spiroplasma*, now known as *S. ixodetis* (*6*), and a systemic infection in an immunocompromised adult with *S. turonicum* (*7*). These reports suggest that tetracyclines and macrolides are effective against *Spiroplasma* (*6*,*7*). We describe 3 newborns in France who had cataract and intraocular inflammation and in whom *S. ixodetis* was detected in ocular samples (Table).

**Table:** Characteristics of 3 newborns with cataract and anterior uveitis* and 5 controls with congenital cataracts without signs of intraocular infection, France†

ID no.	Sex	Date of diagnosis	Age at diagnosis/lens extraction, mo	Affected eye	Clinical ocular findings	Region of residence (environment)	Travel during pregnancy	Crystalline lens sample volume, μL	Bacterial 16S-rRNA PCR, % homology to *S. ixodetis*
Case-patients									
1	F	2014 Jan	1/2	Both	Cataract + anterior uveitis	Hauts-de-France, France (rural area, adjacent to Saint-Gobain Forest)	No	200	98.7
2	M	2018 Jan	0/3	Both	Cataract + anterior uveitis	Centre-Val de Loire, France (rural area, adjacent to Loire-Anjou-Touraine Regional Forest)	No	150	98.6
3	M	2019 Jan	1/2	Left	Cataract + anterior uveitis	Ile-de-France, France (Paris suburban area)	No	100	98.7
Controls									
1	F	2018 Feb	4/5	Right	Cataract	NA	NA	50	Negative
2	M	2018 Apr	4/5	Right	Cataract + nystagmus	NA	NA	10	Negative
3	M	2018 Apr	4/5	Left	Cataract + nystagmus	NA	NA	200	Negative
4	F	2018 Feb	1/2	Left	Cataract	NA	NA	100	Negative
5	F	2018 Mar	0/3	Left	Cataract	NA	NA	50	Negative

*Positive for *Spiroplasma ixodetis* in crystalline lens material by bacterial 16S-rRNA PCR. †For all case-patients and controls, pregnancy was normal and delivery was uneventful. NA, not applicable.

## The Case-Patients

**Figure 1:**
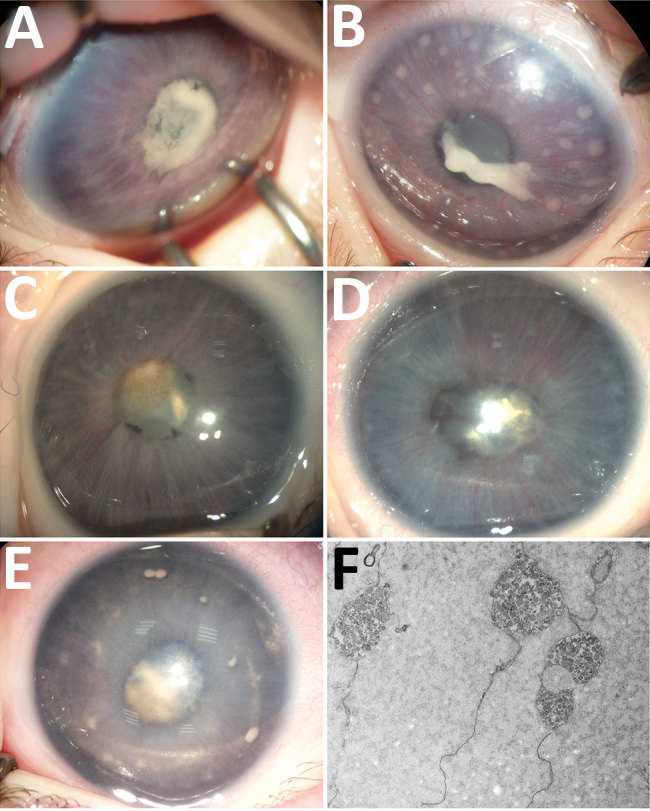
Ocular anterior segment in 3 newborn infants with bilateral total cataract and anterior uveitis related to endogenous *Spiroplasma ixodetis* infection. A, B) Case-patient 1. Right (A) and left (B) eyes of a 4-week-old girl showing total cataract, posterior synechiae due to a cyclitic fibrinic membrane, and large keratic precipitates more visible in the left eye. The immature iris vasculature is dilated in the context of anterior segment inflammation. C, D) Case-patient 2. Right (C) and left (D) eyes of a 6-week-old boy showing total cataract, posterior synechiae, dilated immature iris vessels, and few keratic precipitates more visible in the left eye. E) Case-patient 3. Left eye of a 1-month-old boy with multiple retrocorneal white deposits, total cataract, posterior synechiae, and immature dilated iris vessels. F) Case-patient 3. Electron transmission microscopy of crystalline lens material from a 2-month-old boy with total cataract and anterior uveitis, revealing the presence of microorganisms with spiral-like projections highly suggestive of bacteria from the *Spiroplasma* genus.

**Figure 2:**
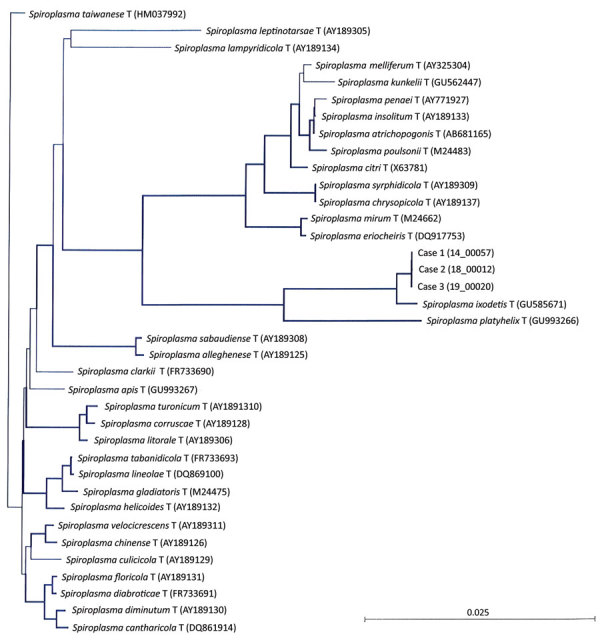
Neighbor-joining unrooted tree based on bacterial *rrs* gene sequences from the crystalline lens samples from 3 newborns with cataract and uveitis (case-patient 1), sample 14_00057; case-patient 2, sample 18_00012; case-patient 3, sample 19_00020). The 14_00057 (case-patient 1 and 19_00020 (case-patient 3) sequences differed by 1 nt along the 1,284-bp bacterial *rrs* gene, and the 18_00012 sequence (case-patient 2) harbored 2 additional nucleotides and differed from 14_00057 by 3 nt and from 19_00020 by 2 nt. At the variation site, the corresponding sequences were _ _ G (14_00057, case-patient 1), T G A (18_00012, case-patient 2), and _ _ A (19_00020, case-patient 3). Thick lines indicate bootstrap values >75% (based on 1,000 replicates). Scale bar indicates the proportion of substitutions per nucleotide position.

In January 2014, case-patient 1, a 26-day-old girl with unremarkable medical history, was referred for bilateral leukocoria observed by her parents at 20 days of age, suggestive of retinoblastoma. She was born after normal full-term pregnancy without delivery complications (birthweight 2,820 g; Apgar score 10). She had bilateral anterior uveitis, large keratic precipitates, iris nodules, posterior synechiae, cyclitic membrane, and cataract (Figure 1, panels A, B). Fundus visualization and ocular ultrasonography ruled out retinoblastoma. Physical examination results were unremarkable. Blood cell count showed elevated monocytes (1.5 × 10^9^/L [reference range 0.2–1.0 × 10^9^/L]); serologic results for *Toxoplasma gondii*, rubella virus, cytomegalovirus, herpes simplex viruses 1 and 2, HIV-1 and -2, and *Mycoplasma* were negative. Aqueous humor cytologic examination did not reveal malignant cells but identified macrophages, suggesting intraocular infection, as observed in *Tropheryma whipplei*–related uveitis (*8*). Treatment with topical dexamethasone (8 drops/d with progressive tapering), topical atropine (0.3%, 2 drops/d), and oral josamycin (125 mg 2×/d) was initiated. Anterior chamber inflammation decreased dramatically, and cataract surgery with intraocular lens implantation was performed sequentially in both eyes 4 weeks later. We conducted microbiological investigations of lens and anterior vitreous aspirates from the right eye, including bacteriologic and mycologic cultures, and 16S-rRNA-based PCR for bacterial identification (Appendix). Cultures remained negative, but bacterial PCR identified a complete sequence of the *rrs* gene, showing 98.7% similarity to the type strains of *S. ixodetis* (Figure 2). Uveitis did not recur over the next 4 years.

In January 2018, bilateral leukocoria caused by bilateral congenital cataract was detected in an otherwise healthy boy, case-patient 2, on day 3 after full-term birth (birthweight 2,900 g; Apgar score 10). Pregnancy was unremarkable, without maternal seroconversion for toxoplasmosis, rubella, herpes simplex viruses 1 and 2, or cytomegalovirus. Six weeks after birth, ophthalmologic examination under anesthesia revealed a total cataract in each eye with large keratic precipitates, posterior synechiae, and immature dilated iris vessels (Figure 1, panels C, D). Fundus was inaccessible in both eyes. The right eye was slightly microphthalmic. Because of the rarity of uveitis with keratic precipitates and cataract in newborns and the similarity to case-patient 1, the child underwent bilateral cataract extraction without lens implantation, and lens material was sent for bacteriologic investigations. The 16S-rRNA–based PCR of the *rrs* gene identified in both eyes was 98.6% similar to *S. ixodetis*. Mild, self-resolving bilateral intravitreal hemorrhage developed after cataract surgery. Anterior segment inflammation resolved under topical dexamethasone (4 drops/d with progressive tapering over 3 months) and atropine (0.3%, 1 drop/d for 1 month) and did not recur over the next 18 months.

In January 2019, case-patient 3, a 1-month-old boy, was referred for left eye leukocoria, first observed 1 week after birth. Pregnancy was uneventful, and delivery was normal at 36 weeks’ gestation (birthweight 2,800 g; Apgar score 10). Left eye examination revealed multiple large keratic white deposits, total cataract, posterior synechiae, and immature dilated iris vessels (Figure 1, panel E). Results of a right eye examination were unremarkable. He underwent cataract extraction with synechialysis, without intraocular lens implantation. 16S rRNA–based PCR of the bacterial *rrs* gene performed on crystalline lens aspirates identified *S. ixodetis* with 98.7% similarity. Fresh crystalline lens samples analyzed by electron transmission microscopy revealed microorganisms with spiral-like projections matching the morphology of bacteria from the *Spiroplasma* genus (Figure 1, panel F). Postoperative mild intravitreal hemorrhage developed but self-resolved over 4 weeks. He was treated postoperatively with oral josamycin (125 mg 2×/d for 10 days), topical atropine (0.3% 2×/d for 1 month), and topical drops combining neomycin, polymixin B, and dexamethasone (4×/d with progressive tapering over 1 month). Intraocular inflammation did not recur over the next 6 months.

We conducted all PCRs in *Spiroplasma* DNA–free facilities. Internal negative controls were introduced during DNA manipulation/amplification (Appendix). No control was positive after 16S-rRNA PCR amplification, confirming that the detection of *Spiroplasma* sequences did not result from contamination.

To confirm that *S. ixodetis* is absent in intraocular media of newborns with noninflammatory cataracts, we collected crystalline lens samples from 5 newborns with congenital cataracts who underwent surgery before 6 months of age (Table). 16S rRNA–based PCR did not identify any bacterial signature in these samples. The Internal Review Board of the French Society of Ophthalmology approved this study.

## Conclusions

Until recently, *Spiroplasma* spp. were considered nonpathogenic in humans. Our observations confirm the reports by Lorenz et al. of an intraocular *Spiroplasma* spp. infection (*6*), and by Aquilino et al. of a systemic infection (*7*). Moreover, the congenital presentation of case-patients 1–3 suggests maternal–fetal transmission during pregnancy or delivery, despite the absence of maternal symptoms. Our findings are consistent with those of Lorenz et al., who described a premature baby born at 27 weeks’ gestation who, at 4 months of age, had unilateral uveitis with corneal precipitates, posterior synechiae, and cataract. After cataract surgery, bacterial 16S-rRNA PCR of vitreous and lens aspirates identified *Spiroplasma* spp. Group VI (*6*), now referred to as *S. ixodetis* (*9*). Electron microscopy visualized filamentous and helical microorganisms compatible with *Spiroplasma*.

Another clade of *Spiroplasma*, *S. mirum*, phylogenetically close to *S. ixodetis* (Figure 2), initially named suckling mouse cataract agent (*9*,*10*), induces rapid cataract formation after intracerebral injection in newborn mice (*11*), rats (*12*), and rabbits (*13*), with variable intraocular inflammation. In these models, adult animals do not develop ocular pathology, suggesting a vulnerability of the immature eye to *Spiroplasma* infection. Moreover, a high rate of microphthalmia developed in these animals, as in case-patient 2, suggesting that *Spiroplasma* infection might interfere with ocular development.

Our observations suggest that intrauterine or early postnatal contamination with *Spiroplasma* spp. might lead to unilateral or bilateral cataract and anterior uveitis in newborns. A similar causative *S. ixodetis* subtype was identified in all 3 infants, without technical contamination. Two of 3 case-patients lived in a rural area adjacent to a forest. 

The frequency of this intraocular infection in newborns may be underestimated. *Spiroplasma* are fastidious organisms detectable using PCR techniques not routinely performed on intraocular samples. Because affected infants are at high risk for visual impairment or blindness, pediatricians, ophthalmologists, and microbiologists should be aware of possible *S. ixodetis* ocular infections and collect clinical, bacteriologic, and epidemiologic data on this emerging pathogen. The mechanisms and timing of probable maternal–fetal transmission require further investigations. On the basis of these observations, we recommend systematic bacterial 16S-rRNA PCR analysis on intraocular fluids and lens material from neonates with cataract and uveitis.

Appendix Methods for detection of *Spiroplasma ixodetis* in ocular samples.

## References

[1] Gasparich GE. Spiroplasmas: evolution, adaptation and diversity. Front Biosci. 2002;7:d619–40. 11861210 10.2741/A799

[2] Regassa LB, Gasparich GE. Spiroplasmas: evolutionary relationships and biodiversity. Front Biosci. 2006;11:2983–3002. 10.2741/2027 16720370

[3] Cisak E, Wójcik-Fatla A, Zając V, Sawczyn A, Sroka J, Dutkiewicz J. *Spiroplasma* - an emerging arthropod-borne pathogen? Ann Agric Environ Med. 2015;22:589–93. 10.5604/12321966.1185758 26706960

[4] Tully JG, Whitcomb RF, Bove JM, Saglio P. Plant mycoplasmas: serological relation between agents associated with citrus stubborn and corn stunt diseases. Science. 1973;182:827–9. 10.1126/science.182.4114.827 17772158

[5] Whitcomb RF, Tully JG, Bové JM, Saglio P. Spiroplasmas and acholeplasmas: multiplication in insects. Science. 1973;182:1251–3. 10.1126/science.182.4118.1251 4796196

[6] Lorenz B, Schroeder J, Reischl U. First evidence of an endogenous *Spiroplasma* sp. infection in humans manifesting as unilateral cataract associated with anterior uveitis in a premature baby. Graefes Arch Clin Exp Ophthalmol. 2002;240:348–53. 10.1007/s00417-002-0453-3 12073057

[7] Aquilino A, Masiá M, López P, Galiana AJ, Tovar J, Andrés M, et al. First human systemic infection caused by *Spiroplasma.* J Clin Microbiol. 2015;53:719–21. 10.1128/JCM.02841-14 25428150 PMC4298541

[8] Touitou V, Fenollar F, Cassoux N, Merle-Beral H, LeHoang P, Amoura Z, et al. Ocular Whipple’s disease: therapeutic strategy and long-term follow-up. Ophthalmology. 2012;119:1465–9. 10.1016/j.ophtha.2012.01.024 22420960

[9] Gasparich GE, Whitcomb RF, Dodge D, French FE, Glass J, Williamson DL. The genus *Spiroplasma* and its non-helical descendants: phylogenetic classification, correlation with phenotype and roots of the *Mycoplasma mycoides* clade. Int J Syst Evol Microbiol. 2004;54:893–918. 10.1099/ijs.0.02688-0 15143041

[10] Tully JG, Whitcomb RF, Williamson DL, Clark HF. Suckling mouse cataract agent is a helical wall-free prokaryote (*spiroplasma*) pathogenic for vertebrates. Nature. 1976;259:117–20. 10.1038/259117a0 1246348

[11] Olmsted E, Prasad S, Sheffer J, Clark HF, Karzon DT. Ocular lesions induced in C57 mice by the suckling mouse cataract agent (SMCA). Invest Ophthalmol. 1966;5:413–20. 5912547

[12] Friedlaender RP, Barile MF, Kuwabara T, Clark HF. Ocular pathology induced by the suckling mouse cataract agent. Invest Ophthalmol. 1976;15:640–7. 1085291

[13] Kirchhoff H, Heitmann J, Trautwein G. Pathogenicity of *Spiroplasma* sp. strain SMCA in rabbits: clinical, microbiological, and histological aspects. Infect Immun. 1981;33:292–6. 7263065 10.1128/iai.33.1.292-296.1981 PMC350688

