# Supplementary material for: Ocular Spiroplasma ixodetis in Newborns, France
Source: Emerg Infect Dis. 2020 Feb;26(2):340–4. doi: 10.3201/eid2602.191097 (PMC6986854; doi:10.3201/eid2602.191097)
Supplement: Appendix — Methods for detection of Spiroplasma ixodetis in ocular samples. [file 19-1097-Techapp-s1.pdf]

Article DOI: https://doi.org/10.3201/eid2602.191097

Ocular *Spiroplasma ixodetis* in Newborns, France

Appendix

Methods for 16S rRNA PCR Bacterial Identification on Crystalline Lens Aspirates Retrieved during Cataract Extraction Surgery (Cases 1, 2, and 3 and Controls)

After surgery, biologic samples were stored at –80°C until analysis. PCR mixes were prepared in a special laboratory equipped with an airlock and with PCR chambers. DNA extraction was performed in a second laboratory with an airlock and Microbiological Safety Cabinet. Positive amplifications were performed in a third laboratory facility, where *Spiroplasma* spp. samples had never been previously manipulated.

Three negative controls were introduced at key steps of the DNA manipulation and PCR processes: one negative control introduced at the beginning, and one at the end of DNA extraction, respectively, and one negative control introduced in the PCR. None of the three controls was positive for bacterial DNA after 16S-rRNA PCR amplification.

For *rrs* gene (encoding 16S rRNA) sequencing, universal primers were used and 1.5 kb of the *rrs* gene was amplified by PCR (*1*). The amplified product was sequenced with four primers. On one hand, the primer A corresponding to position 8 to 28 of *Escherichia coli* 16S rRNA and the primer rE (5′-GGACTACCAGGGTATCTAAT-3′) corresponds to the complement of positions 806 to 787. On the other hand, the primer D (5′-CAGCAGCCGCGGTAATAC-3′) corresponds to positions 519–536 and the primer rJ (5′-GGTTACCTTGTTACGACTT-3′) corresponds to the complement of positions. Nucleotides were numbered according to Brosius et al., 1978 (*2*). A total of 1446 bp for the *rrs* gene were determined. The complete sequence was compared to all bacterial sequences available from GenBank database using the BLAST program (https://blast.ncbi.nlm.nih.gov/Blast.cgi). A phylogenetic tree was generated with CLC Bio software, using the neighbor-joining algorithm (*3*).

The GenBank accession numbers for *rrs* gene sequences of strains 14–00057 (Case 1), 18–00012 (Case 2) and 19–00020 (Case 3) are MN207005, MN166760, and MN166762, respectively.

Methods for Electron Microscopy Imaging of Crystalline Lens Aspirates Retrieved during Cataract Extraction Surgery (Case-Patient 3)

A fraction of the crystalline lens material retrieved from case-patient 3 was cultured on A7 medium. After 2 weeks, small colonies became visible using a conventional ×4 microscope. The colonies were sampled and resin-included. Seventy-micrometer sections were imaged using transmission electron microscopy to investigate the morphology of the colony-forming microorganisms.

References

1. Janvier M, Grimont PA. The genus *Methylophaga*, a new line of descent within phylogenetic branch gamma of Proteobacteria. Res Microbiol. 1995;146:543–50. PubMed http://dx.doi.org/10.1016/0923-2508(96)80560-2

2. Brosius J, Palmer ML, Kennedy PJ, Noller HF. Complete nucleotide sequence of a 16S ribosomal RNA gene from Escherichia coli. Proc Natl Acad Sci U S A. 1978;75:4801–5. PubMed http://dx.doi.org/10.1073/pnas.75.10.4801

3. Saitou N, Nei M. The neighbor-joining method: a new method for reconstructing phylogenetic trees. Mol Biol Evol. 1987;4:406–25. PubMed
